# Purinergic receptor antagonists inhibit odorant-mediated CREB phosphorylation in sustentacular cells of mouse olfactory epithelium

**DOI:** 10.1186/1471-2202-12-86

**Published:** 2011-08-22

**Authors:** Ruth Dooley, Anastasia Mashukova, Bastian Toetter, Hanns Hatt, Eva M Neuhaus

**Affiliations:** 1 Department of Molecular Medicine, Royal College of Surgeons in Ireland, Beaumont Hospital, Dublin, Ireland; 2 Department of Physiology, College of Medical Sciences, Nova Southeastern University, FL 33328, USA; 3 NeuroScience Research Center, Charité - Universitätsmedizin Berlin, Charitéplatz 1, 10117 Berlin, Germany; 4 Department of Cell Physiology, Universitätsstrasse 150, 44780 Ruhr-University Bochum, Bochum, Germany

## Abstract

**Background:**

Extracellular nucleotides have long been known to play neuromodulatory roles and to be involved in intercellular signalling. In the olfactory system, ATP is released by olfactory neurons, and exogenous ATP can evoke an increase in intracellular calcium concentration in sustentacular cells, the nonneuronal supporting cells of the olfactory epithelium. Here we investigate the hypothesis that olfactory neurons communicate with sustentacular cells via extracellular ATP and purinergic receptor activation.

**Results:**

Here we show that exposure of mice to a mixture of odorants induced a significant increase in the levels of the transcription factor CREB phosphorylated at Ser-133 in the nuclei of both olfactory sensory neurons and sustentacular cells. This activation was dependent on adenylyl cyclase III-mediated olfactory signaling and on activation of P2Y purinergic receptors on sustentacular cells. Purinergic receptor antagonists inhibited odorant-dependent CREB phosphorylation specifically in the nuclei of the sustentacular cells.

**Conclusion:**

Our results point to a possible role for extracellular nucleotides in mediating intercellular communication between the neurons and sustentacular cells of the olfactory epithelium in response to odorant exposure. Maintenance of extracellular ionic gradients and metabolism of noxious chemicals by sustentacular cells may therefore be regulated in an odorant-dependent manner by olfactory sensory neurons.

## Background

Odorant receptors (OR) are G protein-coupled receptors which are expressed in olfactory sensory neurons (OSN) of the mammalian olfactory epithelium (OE) [1-3]. Each OSN expresses only one particular type of OR [4] and a given OR gene is expressed in a small subset of OSNs [5,6]. All neurons expressing a particular receptor converge to a single target in the olfactory bulb [5-7]. A total of 347 putative functional OR genes are found in human [8] and approximately 1000 in mouse [9]. Odorant-specific signal transduction is mediated via the olfactory G protein Gα_olf _[10], adenylyl cyclase type III activation [11], the concomitant cAMP-mediated activation of a cyclic nucleotide-gated (CNG) channel [12-15] and the opening of a Ca^2+ ^gated Cl^- ^channel [16,17]. The OE is made up of 3 main cell types: OSNs, basal cells which maintain the regenerative capacity of the OE [18,19] and glial-like sustentacular supporting cells. It is likely that sustentacular cells, as is the case for other glial subtypes of the nervous system, function not only in the maintenance and support of OSNs but also play a role in intercellular signalling mechanisms.

Extracellular nucleotides have long been known to have neuromodulatory functions and to be involved in cellular signalling [20,21]. In the nervous system, ATP may be released by a number of mechanisms from both neurons and non-neuronal cells. ATP is released from neurons as a cotransmitter via vesicle -mediated exocytosis from synaptic terminals, and from non-neuronal cells either by secretion of vesicles or by calcium-independent mechanisms via plasma membrane nucleotide-transport proteins, connexin or pannexin hemichannels [22]. ATP acts as a signalling molecule by binding to and activating purinergic receptors. P2 purinergic receptors bind primarily adenine and uracil tri- and dinucleotides, and comprise 2 families - ionotropic P2X receptors and G protein coupled P2Y receptors. The P2X receptor family consists of 7 subtypes (P2X_1_-P2X_7_) whereas P2Y receptors comprise at least 8 subtypes (P2Y_1_, P2Y_2_, P2Y_4_, P2Y_6_, P2Y_11_, P2Y_12_, P2Y_13_, P2Y_14_). In the central nervous system, P2X receptors act pre-synaptically to induce neurotransmitter release and P2Y receptors are involved in neuron-glia bidirectional signalling.

Purinergic signalling also plays an important role in peripheral sensory systems such as vision and taste. For example, in the retina, ATP plays diverse roles in neuromodulation, neuron-glia intercellular signalling, retinal development and pathophysiology [21]. It was shown that a flashing light stimulus increased the frequency of calcium transients in Muller glial cells and this effect was blocked by suramin, a purinergic antagonist, as well as apyrase, an ATP hydrolyzing enzyme [23]. Purinergic receptor activation is also involved in taste receptor signalling. In the taste bud, ATP is released as a neurotransmitter and as a paracrine signal for coupling taste cells with differing transduction modalities and glia-sensory cell communication [21]. ATP release from taste-bud type II receptor cells is central to the coding of sweet, bitter and umami taste, acting directly on P2X_2 _and P2X_3 _heteromeric receptors at the chemosensory afferent terminals, and in a P2X2/P2X3 double knockout mouse all gustatory transmission was lost from lingual taste buds [24].

In the olfactory system, OSNs express both ionotropic P2X purinergic receptors and G protein-coupled P2Y receptors on their dendrites, soma and axons. On the other hand, sustentacular cells and basal progenitor cells express only G protein-coupled P2Y receptors, where they are expressed on the cell soma and cytoplasmic extensions of sustentacular cells, and on the basal cell soma located in the basal layer [25]. ATP was shown to modulate the odorant sensitivity of OSNs- activation of purinergic receptors on OSNs evoked inward currents and increases in intracellular calcium, and activation of P2X and P2Y receptors with exogenous or endogenous ATP reduced odor responsiveness [25]. It has therefore been suggested that a constant low level of extracellular ATP exists in the OE which has the ability to induce a tonic suppression of OSN activity [25]. Sustentacular cells also exhibited rapid increases in intracellular calcium in response to purinergic receptor agonists, and this effect was mediated via PLC signalling and the release of calcium from intracellular stores [26]. In the olfactory system, ATP may be released from OSNs and their axons [27,28], sympathethic trigeminal nerve fibres [28], or from cells that are acutely injured by toxic compounds such as high concentrations of odorants [29]. However, in this study we are using a concentration of odorants that was previously shown to induce physiological responses to odorants in the olfactory epithelium in both EOG (electro-olfactogram) recordings and in ratiometric calcium imaging of dissociated olfactory neurons [30].

It was previously shown that odorants also stimulate the rapid activation of the ERK/MAPK pathway, leading to phosphorylation of the cAMP response element binding protein (CREB) and induction of CRE-mediated gene transcription in OSNs *in vivo*, suggesting the possibility that individual OSNs may link odorant detection to gene transcription from CRE-containing promoters [31]. CREB is an inducible transcription factor which is phosphorylated at Ser-133 by a multitude of kinases, whereupon its activation leads to intracellular changes in response to extracellular stimuli [32]. CREB-mediated gene expression has been shown to be necessary for the survival of multiple neuronal subtypes [33-37], and to play a central role in differentiation, synaptic plasticity and memory [32].

In the present study, we hypothesized that olfactory signal transduction in OSNs could induce the release of ATP which could act as an intercellular signaling molecule, allowing OSNs to communicate with their glial counterparts, the sustentacular cells. We found that exposure of mice to a complex mixture of 100 different odorants, in order to activate as many OSNs as possible, led to an increase in phosphorylated MAPK in OSNs and an increase in the levels of phosphorylated CREB in the nuclei of both OSNs and sustentacular cells of the olfactory epithelium. The activation of CREB in sustentacular cells was dependent on ACIII-mediated olfactory signaling and purinergic receptor signaling. Odorant-mediated signaling events may therefore be linked to CRE-mediated transcription not only in OSNs themselves but also in neighbouring supporting cells, the sustentacular cells.

## Results

### Odorants induce CREB phosphorylation in the olfactory epithelium

**Figure 1:**
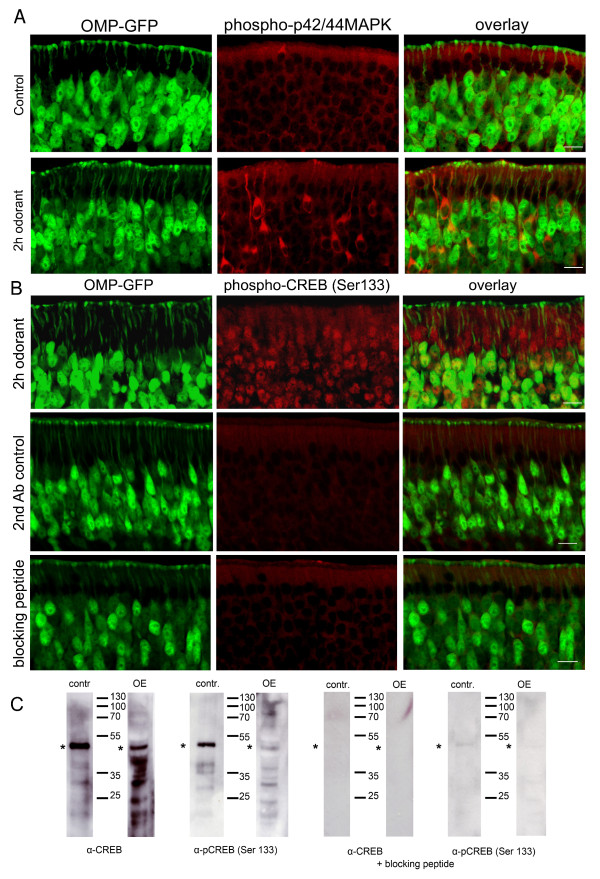
**Odorants induce MAPK and CREB phosphorylation in the olfactory epithelium**. (A) Cryosections (12 μm) of mice expressing GFP in every mature OSN (OMP-GFP) were subjected to immunohistochemistry using an antibody specific for phosphorylated p42/44 MAPK in the olfactory epithelium. After 2 hours of odorant exposure a strong anti-phospho-p42/44 MAPK immunoreactivity was observed in the cytoplasm of OSNs. (B) Staining for phospho-CREB (Ser-133). Phospho-CREB was observed in the sustentacular cell layer, in the nuclei of OSNs, and in immature OSNs not expressing OMP, after exposure to a mixture of 100 different odorants for 2 h. Control panel with secondary antibody only or after using a specific blocking peptide shows very low background labeling intensity in the nuclei of the different cell types. (C) WB analysis of control cell lysates (SK-N-MC cells prepared with IBMX and forskolin treatment) and olfactory epithelium samples with CREB and phospho-CREB (Ser-133) specific antibodies showing labeling of the proteins (bands marked by stars), which is abolished when the specific blocking peptide was pre-incubated with the antibody.

The cAMP response element binding protein (CREB) is a well-characterized transcription factor which is activated upon phosphorylation at residue Ser-133, an event induced by various extracellular signals [32]. Odorants induce the activation of the ERK/MAPK pathway, leading to phosphorylation of CREB in OSNs *in vitro*, after short-term odorant treatment [31]. We have previously shown that β-arrestin, which can function as a molecular scaffold recruiting components of the MAPK cascade, accumulated in intracellular vesicular structures in OSNs after prolonged odorant treatment of a 2 hour duration [30]. Therefore we examined the activation of MAPK under the same conditions using antibodies specific to phosphorylated MAPK (Figure 1A). β-arrestin stably interacts with recombinant olfactory receptors and is evident in endocytic structures following odorant treatment, therefore one could speculate that the olfactory receptor may also be present in these vesicular structures. Stable intracellular GPCR/β-arrestin complexes are involved in the initiation and/or regulation of other signaling pathways.

Here we show that p42/44 MAPK phosphorylation occurs in the olfactory epithelia of OMP-GFP mice (expressing GFP in every mature olfactory sensory neuron) [38]*in vivo *upon OR activation. We exposed mice to a complex mixture of 100 different odorants (Henkel 100). This mixture includes aromatic and short-chain aliphatic hydrocarbons [39,40], and is used in order to activate a large number of olfactory sensory neurons, each expressing different olfactory receptors. Exposing mice to a concentration of this odorant mix that was previously shown to evoke physiological responses in the OE [30], we observed an increase in the phosphorylation of p42/44 MAPK in the cytosol of OSNs, which was not observed in neurons of untreated mice (Figure 1A). We next examined CREB activation, by using an antibody specific to CREB phosphorylated at Ser-133, and found induction of CREB phosphorylation not only in mature olfactory sensory neurons, but also in the sustentacular cells and immature neurons not expressing OMP-GFP (Figure 1B). The specificity of both total CREB and phospho-CREB antibodies was demonstrated using a specific blocking peptide (Figure 1C).

**Figure 2:**
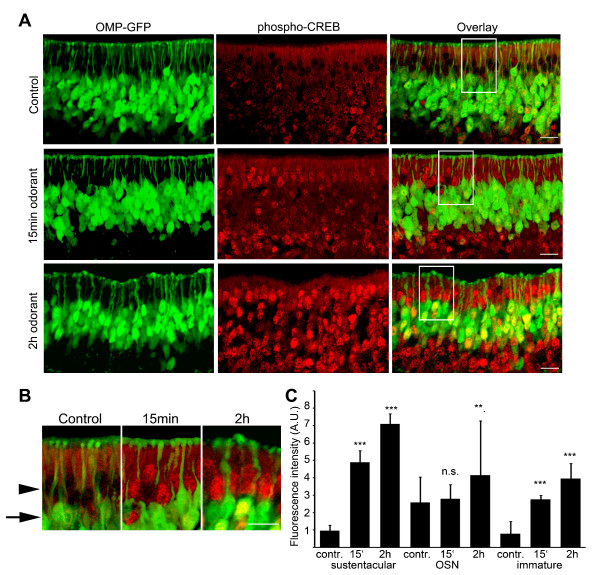
**Time-dependent odorant induced CREB phosphorylation**. (A) Staining for phospho-CREB (Ser-133). Low levels of basal phospho-CREB were observed in the nuclei of OSNs and immature OSNs not expressing OMP, with little or no staining in the sustentacular cell layer. An increase in phospho-CREB in the sustentacular cell layer was observed after 15 min of exposure to a mixture of 100 different odorants, and after 2 hours positive staining for phospho-CREB was observed in the nuclei of OSNs, immature OSNs not expressing OMP and the sustentacular cells. Scale bar 20 μm. (B) High magnification image (2X) of a section of the images in A, clearly showing the increase in CREB phosphorylation in the sustentacular cell layer (arrowhead) and in the OSNs (arrow) after 2 h odorant treatment. Scale bar 20 μm. (C) Bar chart shows mean ± SD average fluorescence intensity along the nuclei of different cell types indicated, normalized to cytosolic background staining. Sustentacular cell nuclei layer, control 0.96 ± 0.33 n = 78, 15 min odorant 4.88 ± 0.68 n = 71, 2 h odorant 7.10 ± 0.59 n = 76. OSN nuclei layer, control 2.58 ± 1.47 n = 68, 15 min odorant 2.80 ± 0.81 n = 81 (p = 0.24), 2 h odorant 4.16 ± 3.11 n = 74. Immature cell nuclei layer, control 0.79 ± 0.69 n = 74, 15 min odorant 2.76 ± 0.24 n = 79, 2 h odorant 3.97 ± 0.86 n = 69. n represents one standard field of view at 40 × magnification and 2 × zoom, one-way ANOVA p*** < 0.001, p** < 0.01. Three mice were used for each condition and four cryosections with complete olfactory epithelium were quantified per mouse.

The increase in CREB phosphorylation was observed after 15 min odorant treatment and increased further after 2 h (Figure 2A, B). Quantification of the fluorescence intensity of the nuclei of the sustentacular cells showed a significant increase in the average fluorescence intensity in the nuclei of sustentacular cells after 15 min and 2 h odorant treatment, as compared to control mice not exposed to any odorants (Figure 2C). Very low levels of CREB phosphorylation were observed in the sustentacular cells of control mice, indicating that CREB activation in these cells is induced by exposure of the olfactory epithelium to odorants. Also the nuclei of the OSNs and the immature sensory neurons (not expressing GFP) showed a significant increase in the average fluorescence intensity after odorant treatment, as compared to control mice (Figure 2C).

### CREB phosphorylation depends on adenylate cyclase signalling

**Figure 3:**
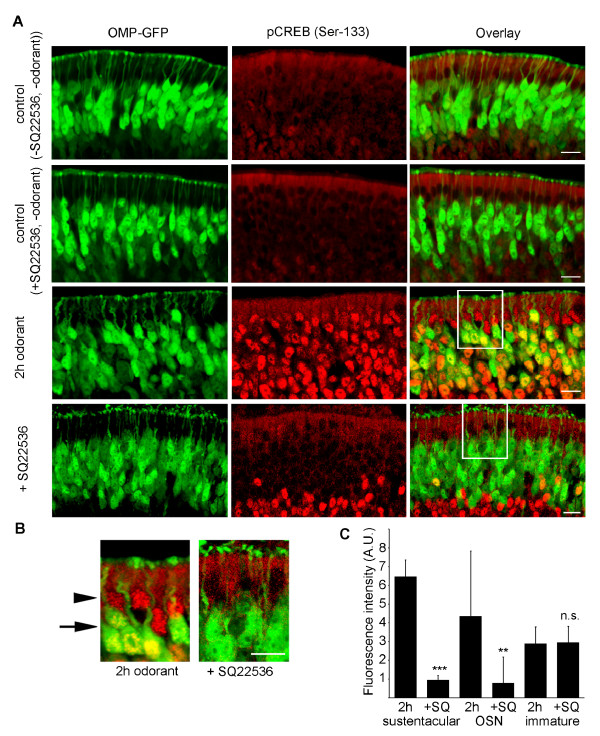
**CREB phosphorylation in sustentacular cells is dependent on ACIII-mediated olfactory signaling**. (A) Cryosections (12 μm) of OMP-GFP mice treated for 2 h with a mixture of 100 odorants or pre-treated with an inhibitor of adenylyl cyclase III (SQ22536). (B) High magnification image (2X) showing the inhibition of CREB phosphorylation in the sustentacular cell layer (arrowhead) and in the OSN cell soma (arrow), by an inhibitor of ACIII. Scale bar 10 μm. (C) Mean ± SD fluorescence intensity across the nuclei was quantified as before. Sustentacular cells showed a significant decrease from (6.47 ± 0.90, n = 79) after 2 h odorant treatment to (0.95 ± 0.24, n = 72) with SQ22536 pre-treatment; OSN nuclei decreased from 4.36 ± 3.47 (n = 70) after 2 h to 0.80 ± 1.39 (n = 81) with SQ22536 pre-treatment; immature cell nuclei did not change (2.90 ± 0.89 after 2 h (n = 80), 2.95 ± 0.86 (n = 74) with SQ22536 pre-treatment); p** < 0.01, p*** < 0.001.

To examine whether CREB phosphorylation was mediated via adenylate cyclase signaling, the septal bone with the intact olfactory epithelium was carefully removed and pre-incubated with SQ22536, an inhibitor of adenylyl cyclase III (ACIII), a central enzyme in the olfactory signal transduction cascade. By inhibiting the canonical olfactory signal transduction pathway, little or no CREB phosphorylation was observed in the OSNs (Figure 3A-C), which was expected, but also in the sustentacular cells of the olfactory epithelium (Figure 3A-C). A significant decrease in CREB phosphorylation, as quantified by nuclear fluorescence intensity measurements was observed in the sustentacular cell layer after pre-treatment with the ACIII inhibitor as compared to 2 h odorant treatment alone. Interestingly, no reduction in phosphorylated CREB was observed in the immature OSNs not expressing OMP-GFP following pretreatment with the ACIII inhibitor, indicating that canonical olfactory signal transduction is not required for activation of CREB in these cells to take place.

### CREB phosphorylation in sustentacular cells after 24 h odorant treatment

**Figure 4:**
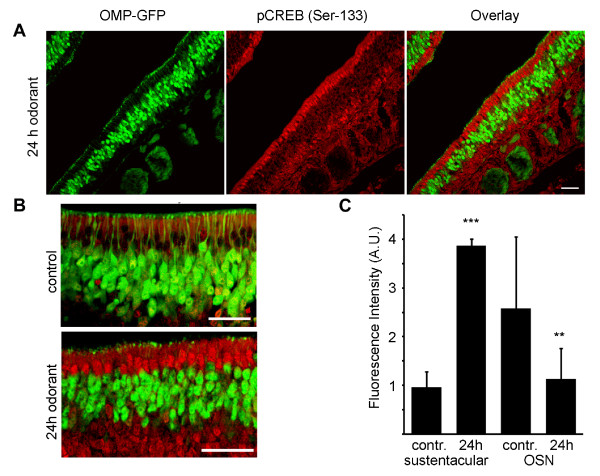
**CREB phosphorylation in sustentacular cells after 24 hours**. (A) Low magnification representative image of 12 μm cryosection of OMP-GFP mice treated with a mixture of 100 odorants for 24 h. Sustentacular cells showed marked CREB phosphorylation, while phosphorylation in immature OSNs not expressing OMP is only weak and in OSNs decreased. (B) Higher magnification images. Scale bars = 50 μm. (C) Fluorescence intensity was quantified as before. Bar chart shows mean ± SD (Sustentacular cell control 0.96 ± 0.33 n = 78, 24 h odorant 3.87 ± 0.13 n = 73; OSN control 2.58 ± 0.15 n = 72, 24 h odorant 1.13 ± 0.62 n = 70), p** < 0.01, p*** < 0.001.

CREB-mediated signaling has previously been implicated in playing a role in the long-lasting effects of odorant exposure in olfactory sensory neurons, for example in the odorant-mediated survival of OSNs by inducing the expression of the anti-apoptotic protein Bcl-2 [41]. We therefore undertook to study CREB activation in the OE after a relatively long period of odorant stimulation, exposing OMP-GFP mice to a mixture of 100 different odorants for 24 hours. Interestingly CREB signalling in sustentacular cells was present even after 24 hours of odorant treatment, as demonstrated by the strong anti-phospho-CREB positive signal in sustentacular cells after 24 h odorant treatment, with little or no phosphorylated CREB in other parts of the olfactory epithelium, including OSNs. Shown in Figure 4 is a low magnification representative image of mouse olfactory epithelium stained with antibodies to phospho-CREB, where strong staining is clearly evident in the nuclei of the sustentacular cell layer. Quantification of the average fluorescence intensity in the layer of sustentacular cell nuclei after 24 h revealed a highly significant increase in phospho-CREB after 24 h, as compared to control mice not subjected to any artificial odor stimulation. In OSNs, a small decrease in CREB phosphorylation was observed after 24 h. This response to 24 h odorant treatment points to long-term trophic effects of olfactory signaling on the supporting sustentacular cells.

### CREB phosphorylation in sustentacular cells is mediated by purinergic receptors

One possible route of intercellular communication between OSNs and sustentacular cells is via extracellular ATP. Sustentacular cells have been shown to functionally express G-protein coupled purinergic receptors [25]. It was previously reported that odorant exposure of olfactory epithelium led to ATP release and the induction of heat shock protein 25 (HSP25) expression in sustentacular cells [29]. We undertook to examine the hypothesis that the activation of purinergic receptors on sustentacular cells by odorant-induced ATP release from OSNs could lead to the phosphorylation of CREB in the sustentacular cells, using physiological concentrations of odorant that were previously shown to mediate specific odorant-mediated activation of OSNs.

**Figure 5:**
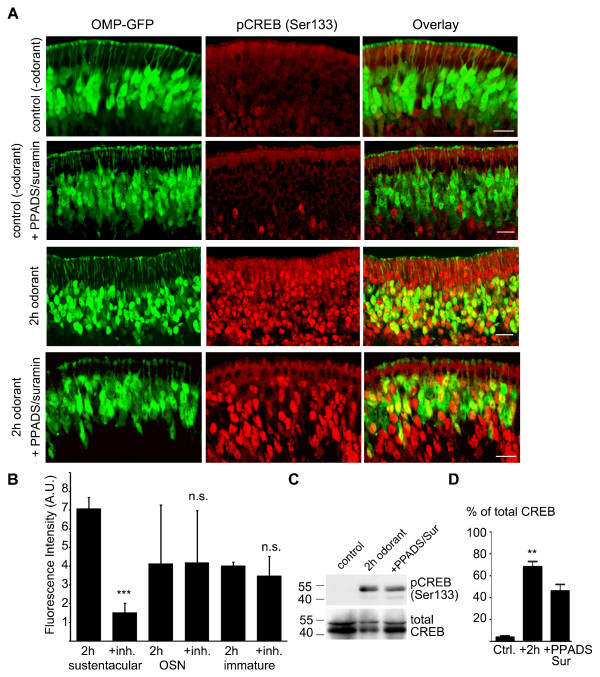
**CREB phosphorylation is dependent on purinergic receptor signaling**. (A) Representative 12 μm cryosection stained with antibodies to pCREB (Ser-133) after pre-treatment with PPADS/Suramin and treatment with 2 h odorant, CREB phosphorylation is markedly reduced in the sustentacular cell layer. Control sections show epithelium without odorant treatment and epithelium without odorant treatment, but with PPADS/Suramin incubation. Scale bar 20 μm. (B) Fluorescence intensities were quantified as before. Bar chart shows mean ± SD. Sustentacular cells 2 h odorant (7.10 ± 0.59 n = 79), +PPADS/Suramin (1.55 ± 0.48 n = 80), OSNs 2 h odorant (4.16 ± 3.11 n = 75), +PPADS/Suramin (4.21 ± 2.76 n = 71, p = 0.48), immature cells 2 h odorant (4.03 ± 2.04 n = 79), +PPADS/Suramin (3.51 ± 1.02 n = 80, p = 0.11), p*** < 0.001. (C) Western blot of whole olfactory epithelia lysates of OMP-GFP mice (2 mice pooled per condition) treated for 2 h with a mixture of 100 odorants or pre-treated with PPADS/Suramin. Blot representative of 3 independent experiments. (D) Bar chart shows average densitometry values, mean ± SEM of phospho-CREB (Ser133), shown as percentage of total CREB. Control (3.70 ± 1.29), 2 hours odorant treatment (68.04 ± 4.98), PPADS/suramin pre-treatment (45.93 ± 6.06), p** < 0.01.

Interestingly, pre-treatment of the olfactory epithelia (the septal bone with the intact olfactory epithelium carefully removed and treated *in vitro*) with a combination of known general purinergic receptor antagonists, 100 μM suramin and 25 μM PPADS, led to a significant reduction in CREB phosphorylation in the sustentacular cell nuclei, as compared to epithelia treated with odorant for 2 h, with no significant decrease in the phosphorylation of CREB in the OSNs or immature OSNs not expressing OMP (Figure 5A, B). These results were confirmed by Western blotting (Figure 5C, D), which revealed a significant increase in CREB phosphorylation in total olfactory epithelium after 2 h odorant treatment, which was reduced after pre-incubation with 100 μM suramin and 25 μM PPADS. This further confirms that purinergic receptor signaling is involved in the induction of CREB phosphorylation in the olfactory epithelium.

### ATP-induced CREB phosphorylation in sustentacular cells is ACIII-dependent

**Figure 6:**
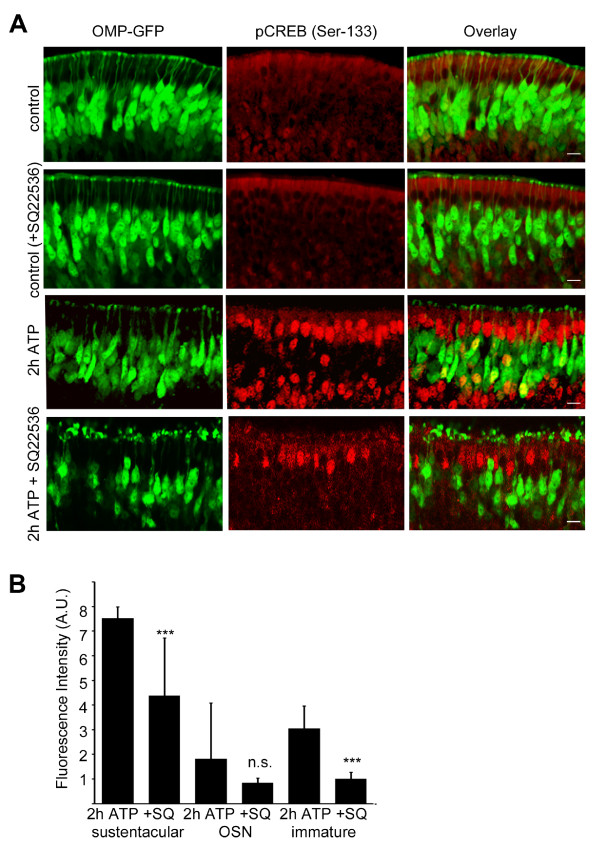
**ATP-mediated sustentacular cell CREB phosphoryation is dependent on adenylyl cyclase III**. (A) Olfactory epithelia were incubated with ATP to activate purinergic receptors, resulting in CREB phosphorylation in sustentacular cells, OSNs, and immature OSNs not expressing OMP. Using an inhibitor to ACIII blocked the phosphorylation of CREB in OSNs, immature OSNs not expressing OMP, and in sustentacular cells to different extents. (B) Fluorescence intensities were quantified as before. Bar chart shows mean ± SD. Sustentacular cells 2 h ATP (7.52 ± 0.47 n = 78), +SQ22536 (4.39 ± 0.24 n = 78), OSNs 2 h ATP (1.81 ± 2.27 n = 75), +SQ22536 (0.84 ± 0.19 n = 69, p = 0.18), immature cells 2 h ATP (3.05 ± 0.91 n = 79), +SQ22536 (1.01 ± 0.27 n = 80), p*** < 0.001. Scale bar 10 μm.

Sustentacular cells were previously reported to express P2Y_2 _receptors [25]. These receptors respond to a variety of ligands, including ATP. On incubation with a solution of ATP (100 μM) we observed an induction of CREB phosphorylation in the olfactory epithelium, in the sustentacular cell layer, in OSNs and also in immature OSNs not expressing OMP-GFP (Figure 6).

Purinergic receptors induce downstream signaling mainly via PLC and IP3. However, some purinergic receptors may signal via adenylyl cyclase and cAMP. Therefore, it was pertinent to ensure the ATP-mediated purinergic receptor signaling in sustentacular cells was not inhibited by the ACIII inhibitor used to block olfactory signaling. After pre-treatment wit h an inhibitor of ACIII (*in vitro*), in the absence of any odorant treatment and in the presence of 100 μM ATP, the phosphorylation of CREB remained evident in the sustentacular cell layer (Figure 6), albeit at a significantly reduced level. This indicates that ATP induces CREB phosphorylation in sustentacular cells to a certain extent via ACIII signaling. Interestingly, inhibiting ACIII blocked the activation of CREB in immature olfactory sensory neurons not expressing OMP-GFP, pointing to a pathway of ATP-mediated activation of CREB in these cells that is entirely dependent on ACIII signaling.

In summary, odorant-induced OSN signaling leads to long-term activation of CREB in neighbouring sustentacular cells, pointing to an important modulatory role of sustentacular cells in the response to chronic odorant treatment. This CREB activation was shown to be purinergic receptor-dependent and therefore implicates extracellular nucleotides in mediating communication between OSNs and sustentacular cells.

## Discussion

Sustentacular cells provide both structural and metabolic support for the olfactory sensory neurons of the olfactory epithelium. These glial-like supporting cells have a multitude of functions including the physical and chemical insulation of OSNs [42], active phagocytosis of dead cells [43], and the regulation of the extracellular ionic environment [42,44,45]. Here we report the induction of phosphorylation of the transcription factor CREB in sustentacular cell nuclei in response to odorant-mediated signal transduction in OSNs. Interestingly, we found CREB activation in sustentacular cells to be inhibited by pre-incubation with purinergic receptor antagonists, suggesting the involvement of extracellular nucleotides in mediating intercellular communication between OSNs and their neighbouring counterparts, the sustentacular cells.

The concept that stimulus detection from extracellular signals, such as hormones, growth factors, and neuronal activity, modulates transcriptional events to produce long-term changes is well established [46]. In many cases changes in transcription occur through the reversible phosphorylation of transcription factors [47-49]. The transcription factor CREB can be activated by many different pathways, such as the neurotransmitter-mediated PKA pathway, calcium-calmodulin-dependent kinases, or growth factor-sensitive kinase pathways [50-52]. CREB is known to be activated by phosphorylation at Ser-133 and in this study we used an antibody specific to CREB phosphorylated at Ser-133 to investigate the activation of this transcription factor.

To date, CREB activation in OSNs via odorant stimulation has already been examined to a certain extent. For example, it has been shown that ERK phosphorylation and CRE-mediated transcription occurred in OSNs in response to short-term treatment with the odorant citralva [31]. Moreover, odorant exposure stimulated the survival of OSNs in an *in vivo *model of apoptosis through the activation of MAPK and CREB [41]. In the present study, we show that exposure of mice to a mixture of 100 different odorants leads to an increase in the levels of p42/44 MAPK phosphorylation in OSNs *in vivo*. Interestingly, CREB phosphorylation occurred not only in OSNs, as was observed for MAPK activation, but also in the sustentacular cells of the olfactory epithelium. The induction of CREB phosphorylation in sustentacular cells was significantly reduced on inhibition of the canonical olfactory signaling cascade, using an inhibitor of ACIII, an enzyme central to odorant-mediated signal transduction which occurs only in OSNs. Odorant-dependent CREB phosphorylation in sustentacular cells therefore seems to be controlled by an intact signaling cascade in olfactory sensory neurons.

Interestingly, on inhibition of purinergic receptor signaling using general purinergic receptor antagonists, CREB phosphorylation remained evident in the nuclei of OSNs and immature OSNs not expressing OMP, but was reduced in the nuclei of sustentacular cells.

Sustentacular cells express P2Y purinergic receptors, in particular subtype P2Y_2 _[25,29] and they respond to P2R agonists ATP and UTP with increases in intracellular calcium. P2Y receptors belong to a G-protein coupled receptor family, and stimulation of these receptors by ATP activates diverse intracellular signaling pathways, including the activation of PLC, PKC, and CaM kinase [53]. In sustentacular cells, purinergic signaling induces PLC activation resulting in the formation of IP3, leading to the release of Ca^2+ ^from IP3-sensitive Ca^2+ ^stores [26], which could possibly lead to Ca^2+^-mediated CREB activation. Alternatively, the activation of PKC leads to the induction of ERK/MAP kinase signaling pathways and CREB phosphorylation at Ser-133 has been shown to be induced by ERK1/2 [54]. Further work will be undertaken in order to dissect the particular mechanism of CREB activation via purinergic receptors in this system.

P2Y receptors can be activated by ATP, which plays an important role in intercellular communication. In the CNS, ATP has been shown to be activity-dependently released from neurons and is also released from astrocytes. Extracellular ATP can spread to neighboring astrocytes and activate membrane receptors, generating an increase in intracellular Ca^2+ ^concentration. Thus, in the CNS, ATP plays an important role in the communication between neurons and astrocytes [55]. For example, Takasaki *et. al*. found that ATP, through the activation of P2Y receptors, the elevation of intracellular Ca^2+ ^concentration, the activation of CaM kinase and the phosphorylation of CREB, induces the upregulation of BDNF exon IV mRNA synthesis in astrocytes [55]. Moreover, in the olfactory system, ATP has been shown to be released by OSNs and their axons [27,28], and from sympathetic and from trigeminal nerve fibres [56-58] and ATP was shown to modulate the odorant sensitivity of OSNs [25].

We found that induction of CREB phosphorylation in the nuclei of the sustentacular cells was mimicked by exogenous ATP application. Pre-incubation with an inhibitor of ACIII reduced the levels of CREB phosphorylation in the sustentacular cell nuclei, however, this inhibition did not completely abolish the phosphorylation of CREB, indicating that the ATP-induced phosphorylation of CREB in sustentacular cells is partially dependent on ACIII signaling. Most of the 8 functional P2Y receptors identified to date act via G-protein coupling to activate phospholipase C, leading to the production of IP3 and mobilization of Ca^2+ ^from intracellular stores [59]; however, some P2Y receptors couple to adenylate cyclase and increase the production of cAMP [60]. Interestingly, phosphorylated CREB in both mature and immature OSNs induced following ATP treatment was completely blocked by pre-incubation with the inhibitor of ACIII, pointing to a different mechanism of ATP-mediated CREB activation in these cells as compared to sustentacular cells. ATP induced CREB phosphorylation in the nuclei of the immature OSNs was reduced upon inhibition of adenylate cyclase signaling, but the odorant induced CREB phosphorylation was adenylate cyclase independent, indicating that different mechanisms are involved. One of these mechanisms is likely to be the activity-dependent ATP signaling from OSNs, the other mechanism and the signal or transmitter involved is unknown at present.

It has previously been reported that odorant treatment may have a damaging effect, causing cell death and leading to the release of ATP in the OE. However, it has also been shown that odorants can have a protective effect on OSNs, enhancing neuronal survival via MAPK and CREB activation [41]. In this study we used concentrations of odorant previously shown to initiate physiological responses in OSNs [30]. Furthermore, the effects we observe in this study are blocked by the specific inhibitor of adenylate cyclase (SQ22536) and therefore are unlikely to be due to unspecific cell death-induced ATP release and more likely to be due to specific olfactory signaling. Therefore, odorant treatment induces the activation of OSNs via a specific olfactory signal transduction cascade involving ACIII, and this signaling is essential for the purinergic receptor-mediated activation of CREB in sustentacular cells.

## Conclusions

In summary, here we outline a novel mechanism of intercellular communication between olfactory neurons and their supporting counterparts, the sustentacular cells, by induction of CREB phosphorylation in sustentacular cell nuclei, which is dependent on the odorant-mediated activation of OSNs. Purinergic receptor antagonists abolished the phosphorylation of CREB in the sustentacular cell nuclei, pointing to a role for extracellular nucleotides in mediating this intercellular communication. The sustained nature of CREB activation in sustentacular cells in response to odorant-mediated transduction in OSNs points to a role in the long-term adaptation of OSNs to chronic odorant stimuli. What remains to be clarified is the functional significance of the induction of CREB phosphorylation in sustentacular cell nuclei after odorant-dependent signal transduction in OSNs, and in particular the CRE-mediated transcription of which particular genes is induced.

## Methods

### Chemicals

The general purinergic receptor antagonists suramin and pyridoxalphosphate-6-azophenyl-2', 4'-disulfonic acid (PPADS), the adenylate cyclase antagonist SQ22536 and adenosine 5'-triphosphate (ATP) were purchased from Sigma. The following antibodies were used: rabbit polyclonal anti-phospho-CREB Ser-133, rabbit polyclonal anti-CREB, and rabbit polyclonal anti- phosphorylated p44/42 MAPK (all from Cell Signaling Technology), secondary goat anti-rabbit conjugated to Alexa 568 (Molecular Probes), secondary goat anti-rabbit-horse radish peroxidase (Biorad). The (positive) control phosphorylated CREB cell extract is total cell extracts from SK-N-MC cells prepared with IBMX and forskolin treatment (Cell Signaling Technology).

### Odorant exposure and murine tissue preparation

All mice were raised and maintained according to governmental and institutional care instructions. For odorant exposure experiments, OMP-GFP mice, expressing GFP in every mature OSN [38], at post-natal day 20, were exposed to a complex mixture of 100 odorants (Henkel 100), including aromatic and short-chain aliphatic hydrocarbons [39,40], in a 1:10,000 dilution in Ringer's solution (140 mM Glucose, 5 mM KCl, 10 mM HEPES, 2 mM CaCl_2_, 1 mM MgCl_2_, 10 mM Glucose, pH 7.4), for the indicated time periods (15 min, 2 hours or 24 hours). All mice were held in standard cages at room temperature. Each cage was housed inside a customized Perspex glass chamber designed to deliver an isolated air stream, and the exhaust air was extracted directly to a fume cupboard. This apparatus was designed to ensure that the vapor phase concentration of the odorant remained consistent throughout the experiment. All tubes were silicon coated to minimized odorant absorbance. Control mice were housed in a separate room free from artificial odorant stimulation.

For experiments using inhibitors or ATP, OMP-GFP mice [38] at post-natal day 20 were sacrificed by cervical dislocation and the septal bone with the intact olfactory epithelium carefully removed and pre-incubated for 15 min in standard Ringer's solution with either 100 μM suramin and 25 μM PPADS in combination, 100 μM adenylate cyclase antagonist SQ22536, 10 μM ATP or in Ringer's solution alone (control) before incubation with the odorant mixture (Henkel 100). The tissue was then fixed in 3% PFA and cryoprotected in 30% sucrose in Ringer's solution overnight at 4°C, before embedding and freezing in Tissue-Tek (Leica, Germany) for immunohistochemistry.

### Immunohistochemistry

Cryosections (10 μm) of mouse olfactory epithelium were prepared and mounted on Superfrost glass slides (Menzel Glaser). Sections were permeabilized and blocked in 1% cold-water fish skin gelatine (Sigma) containing 0.01% Triton X-100 in PBS (blocking buffer) prior to incubation with anti-phopsho-p44/42 MAPK or anti-phospho CREB (Ser-133) primary antibodies diluted in blocking buffer. Sections were incubated with fluorescently labelled secondary antibodies and mounted in ProLong Antifade (Molecular Probes). All fluorescence images are single plane XY images and were were obtained with a confocal microscope (Zeiss LSM510 Meta) using a 40 × 1.4-numerical aperture objective (pinhole set to one Airey unit) and further processed with Photoshop (Adobe Systems Inc., San Jose, CA).

### Western blotting

OMP-GFP mice [38] at post-natal day 20 were sacrificed and the septal bone with intact olfactory epithelium incubated with a 1:10,000 dilution of Henkel 100 or pre-incubated for 15 min in standard Ringer's solution containing 100 μM suramin and 25 μM PPADS. The olfactory epithelium was then carefully dissected into Ringer's solution containing a cocktail of protease and phosphatase inhibitors (Roche), homogenised and lysed. For each experiment, the olfactory epithelia of 2 mice were pooled for each condition (i.e. 2 × control mice, 2 × odorant-treated, 2 × pre-treated with inhibitor) and the experiment was repeated twice. To standardize gel loading and the relative increase in immunoreactivity induced by odorant application, samples were immunoblotted with anti-phospho-CREB antibody, stripped, and reprobed with anti-CREB antibody.

### Quantification and Statistical Analysis

For fluorescence intensity calculations, using the Zeiss LSM510 image processing software, a line was drawn across each image at the level of the sustentacular, OSN or immature OSN cell nuclei and the average fluorescence intensity across this line was calculated. This was normalized to the average background fluorescence intensity of the cell cytoplasm, as measured using a line of equal length. Three mice were used for each condition and four cryosections with complete olfactory epithelium were quantified per mouse. All images were scanned using identical acquisition settings. Results are plotted as mean ± SD. A Student's *t*-test was used when comparing 2 groups and a one-way ANOVA for more than 2 groups, (***p < 0.001, **p < 0.01). For Western blot experiments, densitometry was used to quantify the change in levels of phospho-CREB following odorant treatment. The values for phospho-CREB were normalized to total CREB and calculated as a percentage increase.

## Abbreviations

OR: olfactory receptor; OSN: olfactory sensory neuron; OE: olfactory epithelium; OMP: olfactory marker protein; CREB: cAMP response element binding protein; AC3: adenylyl cyclase 3.

## Authors' contributions

EMN, RD and AM conceived the study, designed experiments, carried out and analyzed the experiments. BT carried out experiments. HH supervised part of the work. RD and EMN wrote the manuscript. All authors read and approved the final manuscript.
